# Maternal Cardiac Arrest in Severe Preeclampsia at 34 Weeks: Successful Resuscitation, Perimortem Cesarean Section, and Dual Survival

**DOI:** 10.7759/cureus.93471

**Published:** 2025-09-29

**Authors:** Pankaj Deori, Habib Md R Karim, Sekhar J Sharma, Himangshu Malakar, Suvan K Chowdhury, Sristi Kumari, Daisy Hazarika, Namitha M Lal, Rohit Sasidharan

**Affiliations:** 1 Anesthesiology, Critical Care, and Pain Medicine, All India Institute of Medical Sciences, Guwahati, Guwahati, IND; 2 Trauma and Emergency, All India Institute of Medical Sciences, Guwahati, Guwahati, IND; 3 Obstetrics and Gynecology, All India Institute of Medical Sciences, Guwahati, Guwahati, IND; 4 Neonatology, All India Institute of Medical Sciences, Guwahati, Guwahati, IND

**Keywords:** acute pulmonary edema, cardiopulmonary resuscitation, intrapartum maternal cardiac arrest, maternal and neonatal outcome, perimortem cesarean section, preeclampsia-eclampsia

## Abstract

Maternal cardiac arrest is an uncommon, devastating event that requires rapid, coordinated action from multiple specialties to give both mother and baby the best chance of survival. We report a 29-year-old woman at 34 weeks and two days of gestation who came to the emergency department with severe preeclampsia and respiratory distress, eventually leading to cardiac arrest. Immediate cardiopulmonary resuscitation (CPR) was started with left uterine displacement (LUD), and return of spontaneous circulation (ROSC) was achieved after five cycles. A lower-segment cesarean section was performed right away, with the baby delivered 15 minutes after ROSC. The neonate was delivered in a state of cardiac arrest, necessitating immediate resuscitation and subsequent admission to the neonatal intensive care unit (NICU). The mother required invasive mechanical ventilation, antihypertensive therapy, and other supportive care. She was discharged to the ward on the third day and was ultimately discharged without apparent neurological sequelae. This case highlights the critical importance of rapid identification and execution of perimortem cesarean section (PMCS) for maternal cardiac arrest to optimize maternal and neonatal survival outcomes. Although perimortem delivery is ideally undertaken within the first few minutes, this case suggests that decisive clinical judgment and effective multidisciplinary coordination, combined with continued high-quality CPR, can yield favorable outcomes even when delivery occurs after 15 to 20 minutes.

## Introduction

Maternal cardiac arrest, once the fetus has crossed the gestational period of viability, is a critical emergency, as two lives are at risk. It requires rapid, coordinated, and team efforts to optimize outcomes for both the mother and fetus. Current guidelines recommend that perimortem cesarean section (PMCS) be considered in pregnant patients beyond 20 weeks' gestation who do not achieve return of spontaneous circulation (ROSC) within five minutes of arrest despite effective resuscitation efforts [1]. Delivery of the fetus reduces aortocaval compression, thereby improving venous return and cardiac output during cardiopulmonary resuscitation (CPR) [1-3]. Evidence supports that the best maternal and neonatal outcomes are achieved when delivery occurs within five minutes of the onset of cardiac arrest. Early preparation and multidisciplinary coordination facilitate timely PMCS, which is critical given the rapid progression to anoxic brain injury after four minutes of arrest. Adherence to these protocols is essential to maximize survival chances in this high-risk scenario [4].

Although resuscitation guidelines for pregnant women mostly align with standard adult procedures, there are several significant differences, most notably the presence of two patients, the expectant mother and her fetus. Providers need to thoroughly understand the risks and causes of maternal mortality to best prevent and treat cardiac arrest in pregnancy [5].

## Case presentation

We present the case of a 29-year-old woman (G_4_P_2_L_1_A_1_) at 34 weeks and two days of gestation, who entered the emergency room with headache, abdominal pain, and breathlessness that had lasted two days. She had undergone two previous cesarean deliveries and had been diagnosed with gestational hypertension in her second trimester in the present pregnancy and was taking labetalol 100 mg three times daily. At 1:25 pm on arrival, her Glasgow Coma Scale was E2V2M5; blood pressure, 206/110 mmHg; heart rate, 96 per minute; oxygen saturation, 74% on room air; and a respiratory rate of 36 per minute. Noninvasive ventilation was initiated to address her breathing difficulties. Since peripheral intravenous (IV) access was complicated by edema, a right subclavian central venous catheter was inserted under sterile conditions. She was given IV labetalol 20 mg, Lasix 20 mg, a nitroglycerine infusion (starting at five mcg/min and increased to 10 mcg/min), and a loading dose of magnesium sulfate (4 g IV) followed by 1 g/h to address possible eclampsia. A Foley catheter was placed for urine monitoring.

Obstetricians were notified immediately. Thirty-five minutes after her arrival, she became unresponsive and went into cardiac arrest at 2:10 pm. CPR began immediately, following advanced cardiac life support (ACLS) guidelines [6]. Left uterine displacement (LUD) using folded towels as a wedge was maintained throughout, and after five cycles of CPR, ROSC returned. Two doses of 1 mg epinephrine were given during resuscitation. The anesthesia team, neonatal intensive care unit (NICU), and operating room (OR) personnel were prepped for a PMCS should she not revive. The trachea was intubated using a 7-mm cuffed tube, and she was transferred to the OR, adjacent to the emergency department, for an emergency cesarean delivery at 2:20 pm. By the time she was on the OR table, she showed signs of gasping and spontaneous circulation, so 1 mg of midazolam was administered IV. While the staff prepared monitors and sterile drapes, the abdomen was opened with a longitudinal surgical incision by 2:30 pm, and the baby was delivered at 2:35 pm by lower segment transverse incision. The placenta was delivered spontaneously, and then the uterus was closed in a single layer. The newborn was cyanosed, limp, did not cry, and had no breathing effort with an Appearance, Pulse, Grimace, Activity, and Respiration (APGAR) score of zero at one minute. The neonatology team intubated and resuscitated the baby with positive pressure ventilation and chest compression. The APGAR improved to three at five minutes. Cord arterial gas showed pH: 6.82 (normal range 7.25 - 7.28), PCO₂: 120.6 mmHg (normal 48), HCO₃: 11.5 meq/L (normal range 18 - 22.5), and base excess of 15.3 (normal range 3.2 - 5.6). The baby was extubated at 18 minutes of life and started on delivery room continuous positive airway pressure (CPAP) and transferred to the NICU. Post ROSC of the baby, there was transient tachypnea of the newborn and respiratory distress, which settled by eight hours of life.

During surgery, the mother was ventilated mechanically and given IV fentanyl 30 mcg and atracurium 25 mg for relaxation. Isoflurane was used during maintenance of anesthesia with age-adjusted minimum alveolar concentrations ranging from 0.5 to 0.6. Invasive blood pressure monitoring was initiated after ROSC using radial artery cannulation. Her blood pressure ranged from 100/70 to 120/80 mmHg up to delivery. Post-delivery, she received two units of oxytocin as an IV bolus, followed by 10 units in 500 mL of Ringer’s lactate solution administered by slow infusion. Her initial blood pressure and heart rate rose briefly after ROSC but stabilized at 120/80 mmHg and a heart rate in the 80s. Peripheral oxyhemoglobin saturation (SpO_2_), which was 65% after ROSC, rose to 85% within ten minutes on 100% FiO_2_ and a positive end-expiratory pressure (PEEP) of 7 cmH_2_O. After delivery, her SpO_2_ stayed at 85-87%. Meanwhile, reversible ‘5H and 5T’s as causes were also evaluated. Arterial blood gas analysis, performed immediately after ROSC, revealed mixed respiratory and metabolic acidosis, as well as hyperglycemia (Table 1).

**Table 1:** Blood gas and metabolic profile of the mother immediately at return of spontaneous circulation. PCO_2_: partial pressure of carbon dioxide; PO_2_: partial pressure of oxygen; SO_2_: saturation of oxygen

Test	Results	Normal value/Range
pH	7.103	7.4
PCO_2_ (mmHg)	54.2	35.0 – 45.0
PO_2_ (mmHg)	75.1	83.0 - 108.0
SO_2_ (%)	84.8	95.0 - 100.0
Bicarbonate (mmol/L)	16.6	22.0 - 26.0
Hematocrit (%)	39.0	36.0 - 53.0
Calcium (mmol/L)	1.141	1.150 - 1.330
Potassium (mmol/L)	4.37	3.50 - 5.10
Sodium (mmol/L)	134.3	136.0 - 145.0
Chloride (mmol/L)	101.8	98.0 - 107.0
Lactate (mmol/L)	4.99	0.20 - 1.80
Glucose (mmol/L)	8.7	4.10 - 5.60

Postoperatively, she was transferred to the intensive care unit (ICU) and kept on mechanical ventilation. Her blood pressure was now 170/102 mmHg. Labetalol infusion was started at 5 mg/h, and magnesium sulfate was administered as per the maintenance dose. Point-of-care ultrasound confirmed bilateral B-lines. On postoperative day 1, her vital signs improved, and she tolerated pressure-support ventilation. On day 2, she was extubated and clinically stable. On postoperative day 3, she was shifted to the obstetric ward, where she remained slightly irritable for the next three days but showed no apparent neurological deficits.

Meanwhile, the baby was also off CPAP in the NICU and breathing room air. Neonatal jaundice of prematurity (total serum bilirubin 6.79 mg/dl) was noted by 19 hours of life, which remained clinically significant until day 5, and the infant was started on phototherapy on day 5. No features of hypoxic-ischemic encephalopathy or end-organ damage were noted; tone, appearances, and reflexes were normal.

## Discussion

Our case highlights the importance of an immediate team-based approach in managing maternal cardiac arrest. Our multidisciplinary team, comprising emergency physicians, anesthesiologists, obstetricians, neonatologists, and critical care specialists, worked in close coordination, with decisions and subsequent management steps taken collectively under the guidance of a senior leader. It further demonstrates that continuing high-quality CPR until the ROSC and maintaining adequate maternal blood pressure and oxygen supplementation can potentially result in dual survival, even if delivery incision is not achieved within the recommended four-minute window for PMCS.

Cardiac arrest in pregnant women is rare, occurring in roughly one in every 36,000 maternities [7]. The major contributing causes for arrests include heart disease (23%), thromboembolism (16%), epilepsy and stroke (13%), and sepsis (10%) [7]. Preeclampsia as a cause of peripartum cardiac arrest contributes only 2% [7]. Cohen and colleagues found that many physicians lacked a proper understanding of parturient physiology, the need for LUD, modified ACLS protocols, and timely PMCS during maternal resuscitation efforts [8]. The “four-minute rule” suggests neonatal outcomes are best when PMCS procedures are carried out within five minutes of cardiac arrest for pregnancies of 24 weeks or more [9].

Kim and colleagues described a 43-year-old woman at 24 weeks of gestation who collapsed outside her home due to cardiac arrest. CPR, including defibrillation, was performed, and ROSC was noted after 19 minutes. Fetal heart rate was found to be reassuring during post-ROSC monitoring, but the mother had high blood pressure and seizures for two subsequent days, which were controlled using magnesium sulfate. She was continued on beta blockers for underlying heart issues and ultimately had an uncomplicated elective cesarean delivery at 37 weeks and two days of gestation, delivering a healthy, normal-weight baby [10].

In the present case, cardiac arrest lasted through five rounds of CPR, with the baby delivered 25 minutes after the arrest occurred and 15 minutes after ROSC. Even though both mother and newborn showed signs of poor respiratory status and the neonate had an initial poor APGAR, persistent multidisciplinary teamwork and focus on maintaining circulation were critical to both surviving. As peripartum cardiac arrest is rare, many clinicians are unfamiliar with specific interventions for pregnant patients [4]. It often leads to slow decision-making during this critical time, resulting in delayed PMCSs, which impact outcomes [8]. Shifting to the OR rather than doing an onsite PMCS can also delay. A hospital's public announcement, such as code blue alerts, can help reduce response time. Thus, understanding how pregnancy changes the body and how these changes affect cardiac arrest resuscitation protocols is vital for effective CPR [11]. Further, it is critical to understand the role of PMCS in increasing the stroke volume and cardiac output of pregnant women. It relieves the aortocaval compression, causes auto-transfusion, improves preload, and reduces oxygen demand [4]. 

Madjer and colleagues reported on a pregnant woman who underwent urgent surgery for a leg injury and subsequently experienced cardiac arrest due to sudden pulmonary embolism (PE) [12]. Her survival, despite a complicated series of events, trauma, massive PE, cardiac arrest, emergency cesarean section, and significant bleeding, shows the strength of modern medical care delivered by skilled and dedicated teams able to diagnose and intervene rapidly.

Burton and colleagues described cardiac arrest in pregnancy as the first sign of severe left ventricular dysfunction, highlighting the value of extracorporeal membrane oxygenation support in such cases and explaining specific treatment approaches for maternal cardiac arrest in this setting [13].

## Conclusions

Our case demonstrates that coordinated, multidisciplinary management, tailored with modifications to ACLS protocols for pregnancy and rapid PMCS, can significantly improve outcomes in perimortem cardiac arrest. While a four-minute rule for perimortem LSCS is emphasized and needs to be followed as far as possible, our case illustrates that swift decision-making and a multidisciplinary team approach can still lead to a positive outcome when delivering the baby within 15-20 minutes of ongoing CPR.

## References

[1] Vanden Hoek TL, Morrison LJ, Shuster M (2010). Part 12: cardiac arrest in special situations: 2010 American Heart Association Guidelines for Cardiopulmonary Resuscitation and Emergency Cardiovascular Care. Circulation.

[2] Kaur M, Jain R, Gulati A, Taneja A, Sardana S, Grewal A (2021). Case series of perimortem caesarean delivery during maternal cardiac arrest: Our initial experience and audit. J Obstet Anaesth Crit Care.

[3] Abbasi S, Taylor RT (2011). Highlights from the literature. Emerg Med J.

[4] Mathur D, Leong SB (2013). Perimortem caesarean section: rethinking the resuscitation codes?. J Obstet Anaesth Crit Care.

[5] Jeejeebhoy FM, Zelop CM, Lipman S (2015). Cardiac arrest in pregnancy: a scientific statement from the American Heart Association. Circulation.

[6] Panchal AR, Bartos JA, Cabañas JG (2020). Part 3: adult basic and advanced life support: 2020 American Heart Association guidelines for cardiopulmonary resuscitation and emergency cardiovascular care. Circulation.

[7] Lott C, Truhlář A, Alfonzo A (2021). European Resuscitation Council Guidelines 2021: cardiac arrest in special circumstances. Resuscitation.

[8] Cohen SE, Andes LC, Carvalho B (2008). Assessment of knowledge regarding cardiopulmonary resuscitation of pregnant women. Int J Obstet Anesth.

[9] Boyd R, Teece S (2002). Towards evidence based emergency medicine: best BETs from the Manchester Royal Infirmary. Perimortem caesarean section. Emerg Med J.

[10] Kim BR, Kim MY, Kang HS, Shim SS, Kim R (2023). Successful full-term delivery after out-of-hospital cardiac arrest during the second trimester of pregnancy: a case report. Clin Exp Emerg Med.

[11] Jeejeebhoy FM, Morrison LJ (2013). Maternal cardiac arrest: a practical and comprehensive review. Emerg Med Int.

[12] Madjer N, Sherlock D, Labchuk A, Mikrut K (2024). Obstetric cardiac arrest: a case report. Cureus.

[13] Burton A, Ratwatte S, Zalcberg D, Morgan M, Narayan R, Cordina R (2024). Cardiac arrest in pregnancy with successful stabilization and delivery on veno-arterial extracorporeal membrane oxygenation: a case report. Eur Heart J Case Rep.

